# Prognostic Factors for Intracranial Progression in Her-2-Overexpressing Breast Cancer Patients with Brain Metastases as Primary Relapse Site—Real-Life Data

**DOI:** 10.3390/cancers18101659

**Published:** 2026-05-20

**Authors:** Agnieszka Majewska, Tomasz Byrski, Michał Falco

**Affiliations:** 1 Radiation Oncology Department, West Pomeranian Oncology Centre, 71-730 Szczecin, Poland; amajewska@onkologia.szczecin.pl; 2 Department of Oncology and Chemotherapy, Pomeranian Medical University, 70-111 Szczecin, Poland; tomasz.byrski@pum.edu.pl

**Keywords:** breast cancer, Her-2 overexpression, brain metastases

## Abstract

**Background:** Her-2 positive breast cancer is characterised by a high risk of metastases to the central nervous system, which significantly affects prognosis. The aim of this study was to analyse risk factors and treatment outcomes for brain metastases diagnosed at the time of first progression. **Materials and Methods:** A retrospective analysis was conducted on 1226 patients treated between 2010 and 2022. Distant metastases were diagnosed in 186 (15.7%) patients, including 48 (25.8%) with BM at the time of first progression. The impact of clinical factors and treatment on overall survival (OS) and intracranial progression-free survival (PFS) was analysed. **Results:** Patients with BM were significantly younger than other patients with distant metastases (mean 52.4 years vs. 61.1 years, *p* = 0.0001). The most significant prognostic factor for OS was the number of intracranial lesions (*p* = 0.0004). OS did not differ significantly depending on the use of local therapy (local therapy vs. whole-brain radiotherapy) or systemic therapy (observation vs. chemotherapy vs. anti-Her2 treatment). **Conclusions:** The number of brain metastases remains a key prognostic factor and should be taken into account in therapeutic decision-making, which highlights the importance of early identification of high-risk patients and the number of intracranial lesions in treatment selection.

## Simple Summary

Among patients diagnosed with breast cancer, brain metastases (BM) most often occur when the tumour cells overexpress the Her-2 receptor. Approximately 20–40% of patients in this group may experience brain disease progression over their lifetime. A significant percentage of patients experience secondary brain lesions as the first relapse of their cancer. Determining the optimal management for this group of patients impacts the quality and length of life. We present factors influencing treatment selection and overall survival outcomes in patients experiencing brain metastases as the first relapse of their Her-2 overexpressing breast cancer.

## 1. Introduction

Metastatic tumours are the most common type of brain cancer in adults and one of the most serious complications of cancer; they may occur up to 10 times more frequently than primary tumours of the central nervous system [1,2]. Brain metastases (BM) develop in 20–40% of cancer patients, most commonly in those diagnosed with lung, breast, melanoma, kidney, and colorectal cancer [3,4,5].

In breast cancer, the incidence of BM is 10–30% and depends on the biological subtype; it is highest in Her-2 positive and triple negative breast cancer (TNBC), while lowest in ER+/HER2– [6,7]. In Her-2 positive patients, BM may occur early and sometimes represent the first site of recurrence; at the metastatic stage, they were found in approximately 25–40% of patients [8,9].

Following diagnosis of BM, the median overall survival has historically been 4–6 months, whereas in Her-2 positive cohorts treated with multimodal therapy it reached 15–24 months [7,10,11]. Despite therapeutic advances, the blood–brain barrier limits the efficacy of many drugs, and prognosis is determined primarily by clinical factors, including the number of BM, control of extracranial disease, and the availability of local and systemic treatment options [12]. The standard of care includes surgery, stereotactic radiotherapy (SRT) or whole-brain radiotherapy (WBRT), and anti-Her2 therapies [11,12,13].

Given the high risk of central nervous system involvement in Her-2 positive breast cancer, it is essential to analyse risk factors and treatment outcomes, particularly in the context of the occurrence of BM at the time of initial disease spread and their clinical characteristics, in particular the number of intracranial lesions, which may influence the choice of local treatment. This study presents our centre’s experience with a cohort of 1226 patients with Her-2 positive breast cancer treated between 2010 and 2022.

In our work, we focused on the analysis of specific group of patients with BM in terms of the primary tumour (breast cancer) and molecular subtype (Her-2 positive). The literature data on this homogenous group are sparce.

## 2. Materials and Methods

Between 2010 and 2022, radical treatment was performed at the West Pomeranian Oncology Centre in a group of 7877 patients diagnosed with breast cancer. Complete clinical data regarding prognostic factors, treatment administered, and post-treatment follow-up were obtained for a group of 6712 patients (85.21%). In this group, 1226 patients (18.26%) were found to have Her-2 receptor overexpression in their tumour cells. During the treatment and follow-up period, tumour progression in the form of distant metastases (DM) was diagnosed in 186 (15.17%) patients. The survival period ranged from 2 to 183 months (mean 80.4 months, median 50 months). At the time of diagnosis of metastatic disease, BM were found in 48 patients (25.8%), of whom 9 (18.75%) had a recurrence confined solely to the brain. Seven patients (14.6%) were treated palliatively: best supportive care or palliative WBRT with doses of 20 Gy in 5 fractions. This group was excluded from further analysis.

**Figure 1:**
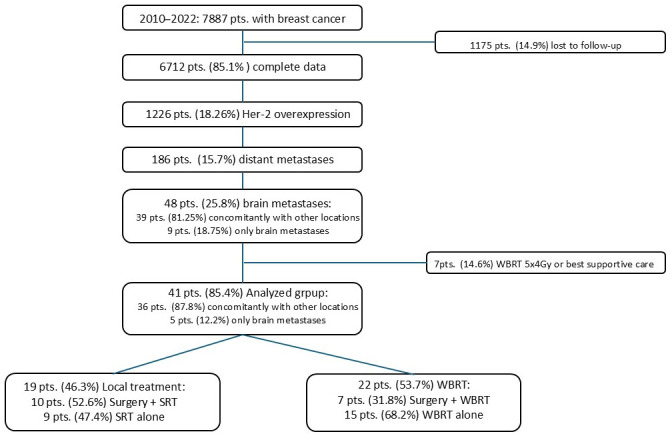
Analysed group summary.

Only 41 (85.4%) patients underwent radiotherapy using SRT or high-dose WBRT. This group of patients was analysed. In total, 19 patients (46.3%) received only local therapy (LT) of BM: 10 received surgery and SRT to tumour bed with regimens 5 × 5 Gy or 5 × 6 Gy, and 9 as standalone SRT in various regimens (2 × 10.5 Gy, 3 × 9 Gy, 5 × 6 Gy). A total of 22 patients (53.7%) received WBRT (7 patients as post-surgery, 15 as standalone WBRT): 16 patients with the dose of 30 Gy in 10 fractions and 6 patients WBRT with a dose escalation in the metastatic area to 40 Gy in 10 fractions. The data presented are summarised in Figure 1.

In total, 10 patients (24.4%) received anti-Her-2 therapy following local treatment of brain metastases (5 patients with lapatinib/capecitabine, 3 patients with docetaxel/trastuzumab/pertuzumab, and 2 with patients trastuzumab emtansine). In this group, there were 3 patients with one BM, 4 with two to nine BM and 2 with more than ten BM (for 1 patient, data on number BM was lacking). Overall, 3 patients (7.3%) received vinorelbine-based chemotherapy alone (2 patients with one BM and 1 with nine BM), while 28 patients (68.3%) did not receive systemic treatment (14 patients with one BM, 7 with two to nine BM, and 7 with more than ten BM).

Student’s *t*-test and the Chi-square test were used to analyse factors influencing the risk of BM (age, time to tumour progression, ER status). A *p*-value of <0.05 was considered statistically significant.

To analyse the factors influencing the choice of treatment for BM (surgery vs. radiotherapy; LT vs. WBRT), the one-way Chi-square test and logistic regression were used. In logistic regression, we used the backward method; the variable was entered when *p*-value was below 0.05 and removed when *p*-value was above 0.1. The model included the following variables: ER status (positive vs. negative), presence of DM outside the brain (yes vs. no), patient’s age (years), time since cancer diagnosis (months), and number of BM (1 vs. 2–9 vs. >10). A *p*-value of <0.05 was considered statistically significant.

Overall survival (OS) was defined as the time from diagnosis of tumour progression to death. Intracranial progression-free survival (PFS) was defined as the time from diagnosis of BM to any progression in the brain. Data on PFS were available only for 29 patients. Kaplan–Meier curves and hazard ratios were used in univariate analysis to calculate PFS and OS. A *p*-value of <0.05 was considered statistically significant.

In the multivariate analysis for OS, we used Cox proportional hazard regression. We used the backward method. The variable was entered when *p* value was below 0.05 and removed when *p* was above 0.1. The following factors were taken into account: use of systemic treatment, number of BM, time of onset of BM, patient’s age (years), ER status (negative vs. positive), and the presence of DM outside the brain (yes vs. no). A *p*-value of <0.05 was considered statistically significant.

Multivariate analysis for PFS was not conducted.

The analysed group is relatively small, which significantly limits the statistical power of the presented results. Our study may be underpowered to detect differences in OS and PFS.

Statistical analyses were performed using MedCalc for Windows, version 23.3.7 (MedCalc Software, Ostend, Belgium).

## 3. Results

The age at diagnosis in the study group ranged from 26 to 88 years (mean 56.6 years, median 55.5 years). The age of patients at the time of recurrence ranged from 28 to 92 years (mean 60.3 years, median 59 years). DM were diagnosed within a period of 1 to 114 months (mean 26.3, median 20). Of the 48 patients with brain metastases at first recurrence, 41 met the criteria for treatment outcome analysis and were included in the further analysis.

The time to onset of BM did not differ significantly from other DM sites (mean 21.6 months vs. 27.9 months, *p* = 0.0942). No statistically significant difference was found in the presence of the oestrogen receptor between the two groups. Age at diagnosis was significantly lower in the group of patients with BM compared to patients with DM in other sites (mean 52.4 years vs. 61.1 years, *p* = 0.0001).

Table 1 presents a summary of the analysis of factors influencing the choice of treatment method. Only the number of BM was a factor significantly influencing the choice of LT (*p* = 0.0374).

In the logistic regression model, only the number of BM proved to be a significant factor in the decision to undergo surgical treatment (*p* = 0.0012; AUC 0.85) and LT (*p* = 0.0446; AUC 0.675). Of the 19 patients with a single BM, 15 (78.9%) underwent surgery. LT was performed in 12 (63.1%) patients with a single BM, five (71.4%) of the 12 patients with 2–9 BM, and only two (28.6%) of the nine patients with 10 or more BM.

**Figure 2:**
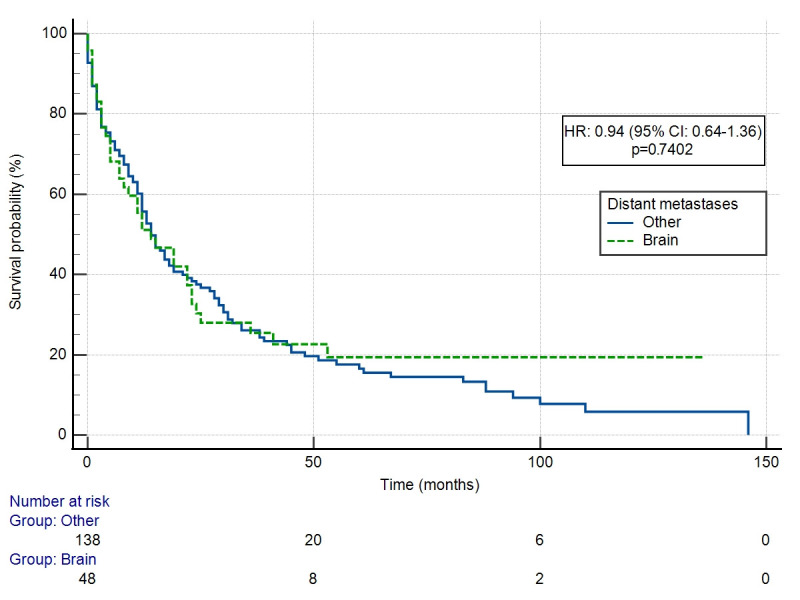
Overall survival depending on distant metastases location.

**Figure 3:**
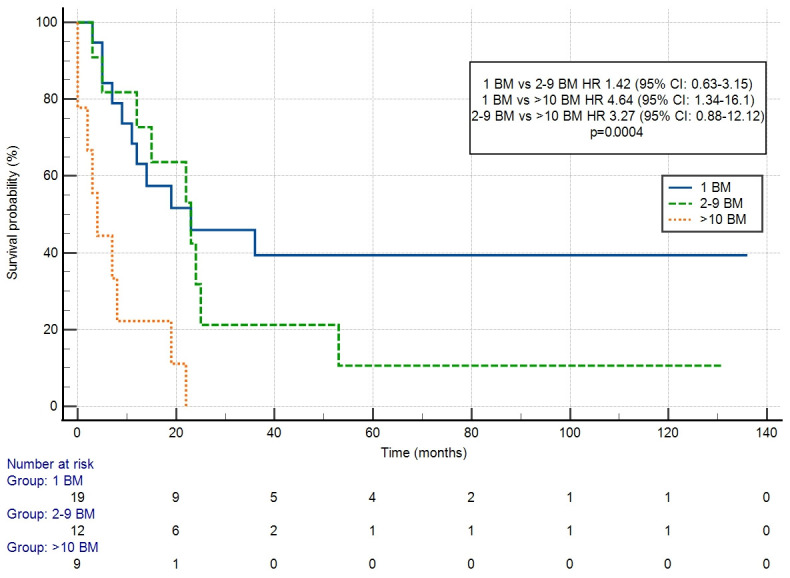
Overall survival depending on the number of brain metastases (BM).

**Figure 4:**
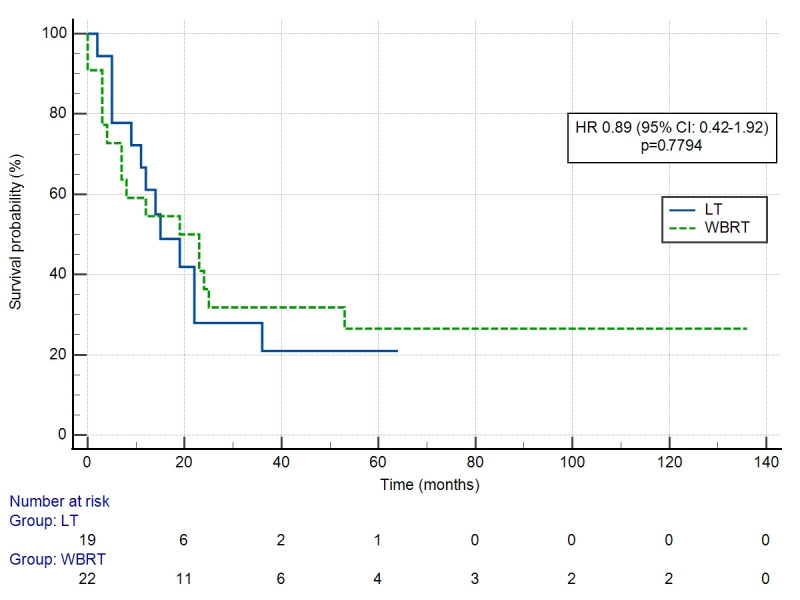
Overall survival depending on brain metastases treatment method (local therapy vs. whole-brain radiation therapy).

A diagnosis of BM did not worsen survival in patients with DM compared to other organ sites (HR: 0.94 (95% CI: 0.64–1.36) (Figure 2). The number of BM significantly influenced OS (*p* = 0.0004) (Figure 3). HR differed significantly only for one versus more than ten BM in comparison (HR 4.64 (95% CI: 1.34–16.1). OS did not differ significantly depending on the use of local treatment (LT vs. WBRT, HR 0.89 (95% CI: 0.42–1.92) (Figure 4) or systemic treatment (observation vs. chemotherapy vs. anti-Her-2 treatment) (*p* = 0.1432). HR for chemotherapy vs. observation was 0.33 (95% CI: 0.05–2.16), while for anti-Her-2 treatment vs. observation, this was 0.73 (95% CI: 0.29–1.81).

**Figure 5:**
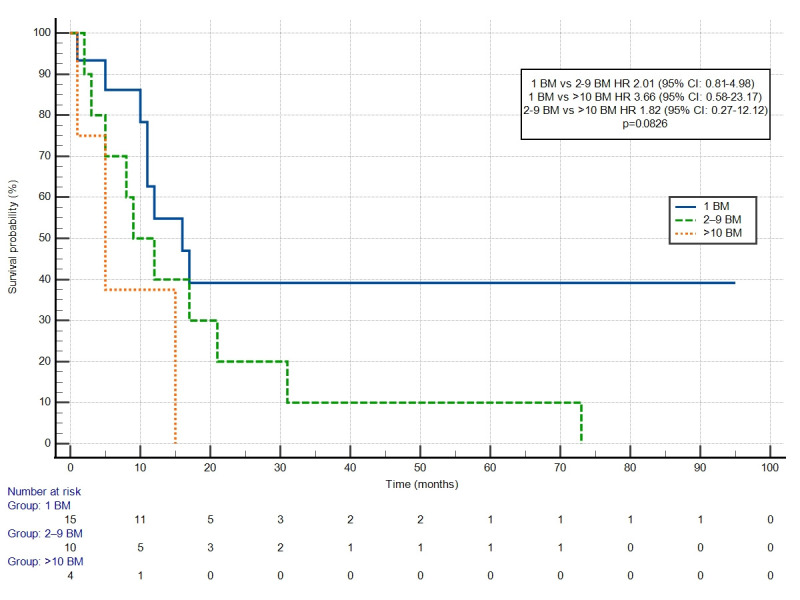
Progression-free survival depending on the number of brain metastases (BM).

The curves for PFS differed depending on the number of BM; however, the difference was not statistically significant: 1 vs. 2–9 HR 2.01 (95% CI: 0.81–4.98); 1 vs. >10 HR 3.66 (95% CI: 0.58–23.17); and 2–9 vs. >10 HR 1.82 (95% CI: 0.27–12.12) (Figure 5). PFS did not differ significantly depending on the local treatment used (LT vs. WBRT; HR 0.52 (95% CI: 0.20–1.35; *p* = 0.1807).

In the Cox multivariate analysis for OS, the only significant prognostic factor was the number of BM (*p* = 0.0017; Harrell’s C-index 0.649, 95% CI: 0.558–0.741) (Table 2).

## 4. Discussion

Her-2 positive breast cancer remains one of the biological subtypes with the highest risk of central nervous system involvement. In our cohort, comprising 1226 patients treated between 2010 and 2022, brain metastases occurred in 25.8% of patients with generalised disease at the time of the first metastasis.

The incidence of BM in our analysis is consistent with the results of large population-based studies and meta-analyses on Her-2 positive breast cancer: Kuksis et al. reported a cumulative incidence of approximately 31% [14]; in the ESME cohort, the figures depended on HR status and follow-up duration—29.2% for HR+/HER2+ and 49.0% for HR–/HER2+ after 24 months [15]; and in a population-based analysis by Wang et al., the cumulative incidence of radiotherapy due to BM was 28.1% for HR+/HER2+ and 34.7% for HR–/HER2+ [16].

Our results therefore confirm that the risk of BM in this group remains high, including when it occurs as the first manifestation of metastasis, despite advances in systemic therapy. Among all patients with DM (*N* = 186), in 48 (25.8%), the first site of metastasis was BM, either alone or with concomitant extracranial lesions. In this subgroup, the proportion of isolated BM (without extracranial lesions) was 18.75% (9/48), which is lower than in most retrospective analyses, where among patients with BM as the first relapse, the proportion of isolated BM was usually within the range of 40–60% [6,9,10]. For the full context, BM with secondary lesions in other locations as the first relapse accounted for 21.0% (39/186) of all cases with distant metastases.

The high proportion of BM may reflect effective control of extracranial disease and the blood–brain barrier effect (“sanctuary”), associated with poor penetration of most anti-Her-2 drugs, which favours the survival of subclinical foci and the emergence of the CNS as the site of first relapse [6,9,17,18].

At the same time, the time to BM onset did not differ significantly from the time to other DM (21.6 vs. 27.9 months; *p* = 0.0942). The lack of statistical significance, despite a trend towards a shorter time to BM, suggests that in some patients, brain involvement may occur as early as other relapses. This points to the coexistence of two mechanisms: early CNS colonisation in patients with greater Her-2 tropism and late manifestation of BM as a result of the above mentioned “sanctuary effect”. Similar observations have been reported in large cohort analyses [15], where the median time to BM did not differ significantly from the median time to other metastases, indicating that in Her-2 positive breast cancer, BM may occur both early and late, depending on tumour biology [15].

We confirmed a significant influence of age on the risk of developing BM—patients with brain involvement were younger at the time of breast cancer diagnosis than those in whom the first metastases appeared in other sites. This finding is supported by numerous studies, where younger age has been identified as an independent risk factor for BM, especially with Her-2 positive and TNBC [7,15,19]. This may be due to the more aggressive biology of tumours in younger women, the more frequent Her-2 overexpression in this group, and the longer survival times during which the risk of BM has time to manifest.

In contrast to some analyses that indicated a higher risk of BM in patients with the Her-2+/ER– subtype [9,15], ER status had no significant impact on the risk of developing BM. This may reflect the heterogeneity of hormonal therapy and the limited size of the cohort.

The most consistent finding from the study is the role of the number of BM. In the univariate analysis, the number of BM significantly influenced OS, and in the multivariate analysis, it remained an independent prognostic factor (*p* = 0.0017). At the same time, the number of BM was the only significant factor influencing the choice of local treatment in the logistic regression analysis, which highlights its key prognostic and decision-making importance, both in relation to surgical treatment and LT vs. WBRT strategy.

The relationship between the number of BM and prognosis is consistent with the DS-GPA data [13], where a higher number of BM is a key determinant of OS regardless of systemic treatment and Her-2 status, and with the RTOG/GPA experience, which—together with the EANO-ESMO guidelines—emphasises the importance of the number of BM, performance status, and control of extracranial disease for prognosis and favours focal treatment when the number of BM is limited [20].

The proportion of WBRT (approx. 53%) and focal treatment (approx. 46%) reflects the evolution of clinical practice over the last decade—from the dominance of WBRT in 2010–2015 to the growing role of focal treatment (SRT) after 2015 [21,22,23]. The structure of the methods used is therefore representative of the period analysed. The analysis of treatment outcomes included a subgroup of 41 patients eligible for local treatment.

In our retrospective cohort, non-significant differences were detected between surgery/SRT and WBRT in terms of OS or PFS. These results should be interpreted with caution. The deviation from the results of randomised trials can be explained by (1) patient selection (mainly patients with fewer lesions were eligible for surgery/SRT); (2) more frequent use of WBRT in patients with multiple BM more aggressive disease; (3) the era of systemic therapies, as following diagnosis of BM, only 24.4% of patients received anti-Her-2 treatment; and (4) the small size of the compared subgroups (LT *n* = 19 vs. WBRT *n* = 22) limiting statistical power. The lack of superiority of any of the local treatment methods should be interpreted in the context of patient selection—surgical treatment was mainly used in patients with a single metastasis, while SRT also included patients with multifocal brain involvement. Such heterogeneity of the groups may have masked potential differences in the efficacy of individual therapeutic strategies.

Randomised trials have shown that adding WBRT to SRT improves PFS but does not prolong OS and impairs cognitive function [21,24,25]. Following resection, SRT of the lesion provides better local control and a more favourable cognitive profile than WBRT [22,26]. Against this background, our data, despite the absence of significant differences, supports the current “lesion-directed” strategy.

Also, in the studies by Niwińska et al. and Le Scodan et al., it was not demonstrated that the timing of BM diagnosis determined the choice of local treatment [7,10]. In our analysis, which included patients with BM as the first manifestation of generalisation, treatment decisions depended primarily on the number of BM, a finding confirmed by the logistic regression model.

In our study, surgical treatment was primarily chosen for patients with single or few (1–2) metastases, and age alone did not influence the decision between surgery and radiotherapy. This pattern is consistent with classic studies and guidelines, which recommend resection mainly for a single, symptomatic lesion with mass effect and good functional status, while for a limited number of lesions, they prefer a focal strategy (SRT or resection + SRT of the bed) over WBRT, due to better local control and neurocognitive profile [21,22,26,27,28,29,30].

Contemporary prognostic scales (RPA/mGPA/DS-GPA) emphasise that the decision is determined more by the number of BM, their size/location, functional status, and control of extracranial disease than by age [13]. Consistent with this is the fact that, in our practice, neither “BM as the first relapse” nor “isolated BM” automatically determined the choice of treatment, but these formed a part of a broader clinical assessment.

Anti-Her-2 treatment following a diagnosis of BM was administered to 24.4% of patients, which corresponds to the results of earlier analyses from the pre-tucatinib and T-DXd era [7,31], where this proportion was limited. In our analysis, the use of systemic therapy did not show a significant impact on overall survival, which may be due to the limited efficacy of the regimens used during the analysed period and the small sample size.

By comparison, modern clinical trials (HER2CLIMB, DESTINY-Breast03, TUXEDO-1) have demonstrated a significant improvement in treatment outcomes, including in patients with BM, which highlights the importance of access to active CNS therapies. For the HER2CLIMB trial, we now emphasise the addition of tucatinib to trastuzumab and capecitabine, highlighting its significant improvement in progression-free survival and overall survival, especially in patients with BM, which is highly relevant to our study’s context. For DESTINY-Breast03, we further detail the superior efficacy of T-DXd compared to T-DM1, including markedly prolonged progression-free survival and higher response rates, establishing T-DXd as a new standard of care in Her-2 positive metastatic breast cancer. For TUXEDO-1, we clarify its importance as a prospective study demonstrating meaningful intracranial activity of T-DXd in patients with active BM, supporting its role in this difficult-to-treat population. These additions provide clearer context regarding current Her-2-targeted therapies and better align our discussion with contemporary clinical evidence [12,32,33]. Our results may not be fully applicable to current clinical practice, with the use of modern anti-Her2 therapies.

We did not observe any significant differences in OS depending on the local or systemic treatment used. The only significant prognostic factor remained the number of BM, which highlights its key clinical importance, particularly in the analysed group of patients with BM as the first manifestation of generalisation.

Some studies suggest a better prognosis for isolated BM with good disease control outside the CNS [7,10]; in our cohort, this effect may not have been apparent due to limited access to active CNS therapies and treatment heterogeneity.

The median time to DM, we observed, was approximately 20 months (mean 26.3 months), while the time to the development of BM was similar (mean 21.6 months; *p* = 0.0942), indicating comparable dynamics in the emergence of lesions in the CNS and other metastatic sites.

These values are not directly comparable with the median OS following the diagnosis of BM reported in retrospective analyses, but they allow for a better understanding of the disease progression in the analysed population.

Despite better control of the disease outside the CNS, brain metastases remain a significant clinical problem. In the APT and KATHERINE studies, the proportion of BM as the first site of recurrence was 2–5% [34,35]. This phenomenon is most commonly explained by the effect of prolonged systemic survival—the longer extracranial disease control is maintained, the more frequently late BM occur.

The high proportion of BM as the first manifestation of dissemination may indicate a specific role of the CNS as a site of recurrence in Her-2 positive breast cancer.

The strengths of the study include: a biologically homogeneous Her-2 positive cohort, analysis of patients with BM as the first site of metastasis, and the use of univariate and multivariate analyses.

Accordingly, the absence of a survival benefit associated with systemic therapy in our cohort should not be interpreted as evidence of lack of efficacy, but rather as a reflection of limited access to effective CNS-penetrant agents and insufficient statistical power.

Despite these limitations, our study provides real-world insight into the natural history and treatment patterns of Her-2 positive BM in a well-defined cohort, including patients with CNS involvement as the first manifestation of metastatic disease. The findings support the central role of intracranial disease burden in prognosis and clinical decision-making.

However, several limitations must be emphasised. These include the retrospective design, potential selection bias, relatively small subgroup sizes, lack of treatment standardisation, and absence of modern systemic therapies. Together, these factors limit the strength and generalisability of the conclusions and underscore the need for cautious interpretation.

## 5. New Fundings and Clinical Implications

In the analysed group a lower percentage of patients (18.75%) with isolated BM as the first DM was observed.

ER status had no effect on the occurrence on BM.

OS did not differ significantly depending on the use of local therapy (LT vs. WBRT) or systemic therapy (observation vs. chemotherapy vs. anti-Her2 treatment).

Our results reinforce that the number of BM remains a key determinant of prognosis and a primary factor guiding local treatment strategies. While our data reflect historical practice, they highlight the importance of integrating focal therapies with contemporary systemic treatments that demonstrate CNS activity.

## 6. Conclusions

In Her-2 positive breast cancer, CNS metastases remain a frequent and clinically significant event, especially in younger patients. The number of intracranial lesions is the most important prognostic factor and a key determinant of treatment selection. Given the limitations of the present study and the rapidly evolving therapeutic landscape, these findings should be considered hypothesis-generating and warrant validation in prospective studies incorporating modern systemic therapies.

## Figures and Tables

**Table 1:** Factors influencing the choice of brain treatment method.

		Operation		*p*-Value	Brain Treatment		*p*-Value
		Yes	No		WBRT	LT	
		N = 17	N = 24		N = 22	N = 19	
Age	Years	53.94 (95% CI: 48.3–59.6)	53.2 (95% CI: 48.28–58.1)	NS	52.2 (95 CI: 47–57.5)	55 (95% CI: 49.9–60.1)	NS
DM time	Months	32.6 (95% CI: 16.1–492)	16.9 (95% CI: 10.2–23.5)	NS	21.2 (95% CI: 9.9–32.5)	26 (95% CI: 14.1–37.9)	NS
ER	Positive	8 (19.5%)	13 (31.7%)	NS	11 (28.8%)	10 (24.4%)	NS
	Negative	9 (21.9%)	11 (28.8%)		11 (28.8%)	9 (21.9%)	
DM	Brain only	2 (4.9%)	3 (7.3%)	NS	2 (4.9%)	3 (7.3%)	NS
	Other	15 (36.6%)	21 (51.2%)		20 (48.8%)	16 (39%)	
No BM	1	15 (37.5%)	4 (10%)	0.0001	7 (17.5%)	12 (30%)	0.0374
	2–9	1 (2.5%)	11 (27.5%)		7 (17.5%)	5 (12.5%)	
	>10	1 (2.5%)	8 (20%)		7 (17.5%)	2 (5%)	

WBRT—whole-brain radiotherapy; LT—local treatment; DM time—time from cancer diagnosis to metastasis; ER—oestrogen receptor; DM—distant metastases; No BM—number of brain metastases.

**Table 2:** Multivariate analysis for overall survival.

Covariate	b	SE	Wald	*p*-Value	Exp (b)	95% CI of Exp (b)
Number of brain metastases	0.8097	0.2576	9.8827	0.0017	2.2472	1.3564–3.7229
Variables not included in the model: Oestrogen receptor—positive vs. negative Age—years Time of onset of BM—months Use systemic treatment—none vs. chemotherapy vs. anti-Her2 treatment						
Overall model fit:						
Null model-2 log likelihood					180.025	
Full model-2 log likelihood					170.332	
Chi-squared					9.693	
DF					1	
Significance level					*p* = 0.0018	
Harrell’s C-index (95% CI)					0.649 (0.558–0.741)	

## Data Availability

The datasets used and/or analysed during the current study are available from the corresponding author on reasonable request.

## Abbreviations

The following abbreviations are used in this manuscript:

DM	Distant metastases
BM	Brain metastases
LT	Local treatment
WBRT	Whole-brain radiation therapy
CNS	Central nervous system
SRT	Stereotactic radiation therapy
PFS	Progression-free survival
OS	Overall survival
